# Detection of *Spirocerca lupi* and an unknown *Trichinella*-like nematode in raccoon (*Procyon lotor*)

**DOI:** 10.1016/j.ijppaw.2024.100911

**Published:** 2024-01-28

**Authors:** Torsten Langner, Domenico Otranto, Marcos Antonio Bezerra-Santos, Jan Franzen, Annette Johne, Daniele Tonanzi, Martin Pfeffer, Stefan Birka

**Affiliations:** a Leipzig University, Faculty of Veterinary Medicine, Institute of Food Hygiene, Germany; b Department of Veterinary Medicine, University of Bari Aldo Moro, Str. prov. per Casamassima km 3, Valenzano, Bari, 70010, Italy; c Institute of Animal Pathology, University of Bern, Bern, Switzerland; d German Federal Institute for Risk Assessment, NRL for *Trichinella*, Max-Dohrn-Str. 8-10, 10589, Berlin, Germany; e European Union Reference Laboratory for Parasites, Istituto Superiore Di Sanità, Rome, Italy; f Institute of Animal Hygiene and Veterinary Public Health, Faculty of Veterinary Medicine, University of Leipzig, An den Tierkliniken 1, 04103, Leipzig, Germany

**Keywords:** *Trichinella*, *Spirocerca lupi*, *Procyon lotor*, Game meat, Foodborne zoonoses, Germany

## Abstract

The raccoon *Procyon lotor* (Carnivora: Procyonidae) is an invasive species of growing importance for the introduction of alien pathogens or as additional hosts for autochthonous pathogens in Europe, including zoonotic parasites. As the population is steadily increasing and outcompeting the red fox (*Vulpes vulpes*) in Germany, the consumption of raccoon meat raises concerns about pathogens they may transmit. Therefore the presence of *Trichinella* larvae was here investigated in muscle samples (n = 904) of raccoons from northern Germany. No *Trichinella* larvae were found, thus confirming the general low occurrence of this parasite in Germany. However, *Spirocerca lupi* (n = 12) and an unidentified *Trichinella*-like nematode (n = 1) were accidently detected in the examined samples. The first is not a zoonotic parasite but has a high veterinary relevance as it can cause severe diseases in dogs. It is the first documented autochthonous infection of this nematode in Germany. The larvae of an unidentified *Trichinella*-like nematode were found in high abundance in all examined muscles of one raccoon, though they could not be identified to species level. Histological investigation revealed intramuscular cystic structures. This is the largest study investigating muscular parasites of raccoons in Europe so far, which suggests that this invasive animal species is infected by *S. lupi* and by a yet unknown *Trichinella* -like parasite.

## Highlights

• Samples of 904 Raccoons were investigated for *Trichinella-*larvae.
• An unknown *Trichinella-*like-Nematode was detected in on individual.
• *Spirocerca lupi* was detected for the first time in Germany.

## Graphical abstract

**Figure:**
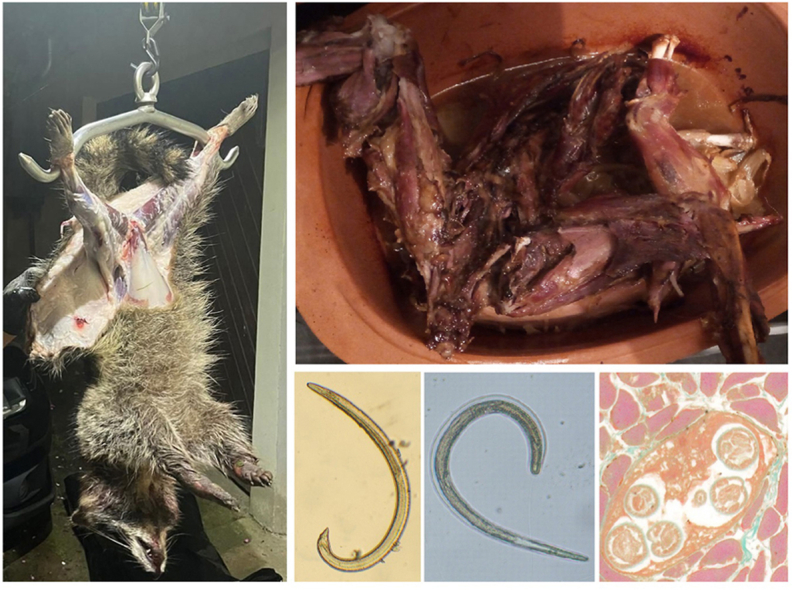

## 1 Introduction

The raccoon (*Procyon lotor*) is a carnivore species from North America which has been introduced to Europe during the last century (Bezerra-Santos et al., 2023). Nowadays this procyonid is well established in Europe (Biedrzycka et al., 2014; Fischer et al., 2015a; Salgado, 2018), with the highest density of population in Germany and the neighboring countries (Beltrán-Beck et al., 2012). Due to the ability to adapt to different environments and the opportunistic omnivore feeding behavior, this animal species is also expanding its geographical range within Germany, and an ongoing spread is expected during the next decades (Fischer et al., 2015b). The raccoon is carrier of a wide spectrum of pathogens, which were either introduced with it (e.g., raccoon roundworm, *Baylisascaris procyonis*) (Rentería-Solís et al., 2018) or are native to Europe (e.g., *Alaria alata* and *Toxoplasma gondii*) (Rentería-Solís et al., 2013; Kornacka et al., 2018) as reported in several countries (Matoba et al., 2006; Azizova, 2015; Sharifdini et al., 2020). Under the above circumstances, raccoons can be a threat to public health due to carrying zoonotic agents (Matoba et al., 2006; Beltrán-Beck et al., 2012; Stope, 2019; Bezerra-Santos et al., 2023). As the number of hunted individuals increases, the possibility to consume their meat is discussed (German Hunting Association, 2020). Reports of restaurants offering this game species (Langner et al., 2022) confirm this consumption of raccoon meat, which potentially represents a source of infection with several zoonotic pathogens. Among others, the raccoon can serve as host for different *Trichinella* species, as reported in invasive populations from Europe and Japan (Kobayashi et al., 2007; Hill et al., 2008; Cybulska et al., 2018), with a single case report in Germany (Langner et al., 2022).

Nematodes of the genus *Trichinella* are causative agent of human trichinellosis, a parasitic disease with worldwide distribution (Gottstein et al., 2009), caused by the consumption of raw or undercooked meat of infected animals. Today, due to high biosecurity level in meat production and official controls in industrial countries, the infection through pork meat consumption is of less concern (Pozio, 2014). Indeed, during the last two decades, the consumption of game meat has been regarded as the most common source of human infection (Pozio, 2001; Rostami et al., 2017; Diaz et al., 2020) and knowledge of the occurrence of this pathogen in the sylvatic cycle is fundamental. For example, specific antibodies against *Trichinella* spp. were detected in raccoon meat juice from Germany (Cybulska et al., 2020), though the examination of this animal species for *Trichinella* spp. is scant.

Analogously, *Spirocerca lupi* is a worldwide distributed nematode with a wide host spectrum, including the raccoon (van der Merwe, Liesel L. et al., 2008; Rojas et al., 2020). The lifecycle includes carnivores as main hosts, coprophagous beetles as intermediate host and rodents, lizards or birds, as paratenic hosts (Rojas et al., 2020). The third stage larvae (L3) which develop and encyst in beetles is the infective stage for the final and paratenic hosts. In the final host larvae develop to adult stage and migrate to mucosa of the esophagus where a tissue reaction is induced, leading to fibro-inflammatory nodules, potentially progressing to metastatic sarcomas (Dvir et al., 2010). The distribution of *S. lupi* is concentrated to warmer tropical and subtropical regions (van der Merwe et al., 2008; Giannelli et al., 2014). This parasite has also been reported in raccoons from Poland, Azerbaijan and Iran (Popiołek et al., 2011; Azizova, 2015; Sharifdini et al., 2020) and eggs were found in faeces samples, suggesting the role of this animal species as definitive host. In order to fill gaps about the parasitic fauna of raccoons in Germany we examined a large number of muscle samples of animals hunted in all areas with occurrence of this game species.

## 2 Material and methods

### 2.1 Sample collection

Raccoons (n = 820) were sampled in a fur producing company which processes hunted carnivores provided by private hunters. Animals included in this study originated from the four fur seasons (15th November to 15th February) 2017/2018 to 2020/2021. Hunters were instructed to mark the carcass with an individual tag provided by the company. Animals with visible fur damages were preselected and excluded and carcasses were stored at −18 °C, until skinning. Carcasses were labeled, weighed, sex determined and the age groups (i. e., juvenile <3.5 months or adult >3.5 months) were assessed based on the presence of permanent canines (Montgomery, 1964). The head, one forelimb, the flexor muscles of the other forelimb, one hindlimb, the gastrocnemius muscle of the other hindlimb and the diaphragm were collected in a plastic bag. All muscle samples were stored in a plastic bag at - 24 °C until used for further examination. Another 84 frozen carcasses of animals hunted in 2020/2021 were provided by a local tannery and stored at −24 °C. Before laboratory examination sex, weight and age determination and sample collection were performed as described above. Information of the geographical origin of these animals was based on the hometown of hunters, though the exact date of killing was not available.

### 2.2 Examination procedures

Frozen muscle samples were defrosted at 5 °C. Before processing, connective tissues and nerves were removed from the samples and samples were cleaned from wood flour (which is used for skinning) under running tap water. Of each animal one half of tongue, one *M. masseter*, one half of diaphragm, 10 g of lower forelimb and 10 g of lower hindlimb were used. Samples of two individuals were assembled to one pool. They were cut into pieces of about 1 cm^3^ and blended (La moulinette 1000W Tefal, Groupe Seb Deutschland, Frankfurt/M.) before the artificial digestion. The digestion of the tissue was performed as described in ISO 18743. However, the digestion time was extended to 120 minutes due to low digestibility of raccoon meat (Malakauskas et al., 2007). The digested material was then sieved as described previously (Franssen et al., 2014). Briefly the liquid was poured through a pile of three sieves with decreasing mesh size. The residues of the first sieve were disposed. The residues of the second and third ones were washed with tap water to the rim and collected in a glass beaker to be microscopically screened following, consecutive filtrations through 300 μm, 180 μm and 63 μm mesh sizes. The collected liquid was dispensed into a modified larvae counting basin (“Trichoview”) for investigation with an inverse stereomicroscope at 40 x magnification as described previously (Makrutzki et al., 2014). In contrast to (Franssen et al., 2014) we used the 63 μm instead of the 38 μm sieve as the smallest one. In case of findings, different muscle sites of included animals were investigated individually the same way, using an adjusted amount of chemicals and water.

Larvae which did not suit the morphology of *Trichinella* were immersed in Amman solution modified (phenol, lactic acid, glycerol, and distilled water in a volume of 1: 1: 4: 1). Measurements and photographs were taken using a light microscope and a Zeiss Zen analysis software for the acquisition and measurement of digital images (Axioscope 5, Carl Zeiss Microscopy GmbH, Jena, Germany).

Muscles of one raccoon infected with nematode larvae were further histologically investigated. Therefore, the skeletal muscle samples from the tongue, diaphragm, flexor muscles of the forelimb, *M. gastrocnemius* and *M. masseter* were fixed for 24 hours in 4 % neutral buffered formalin, embedded in paraffin, cut at 4 μm, and stained with haematoxylin and eosin (H&E), Masson's Trichrome (MT), and Periodic Acid-Schiff (PAS) for further histological evaluation.

For trichinoscopic examination, approximately 0.5 g musculature of the infected individual were split into 28 oat grain sized pieces, pressed between to glass plates and investigated with stereo microscope. *Trichinella* spp. suspected larvae were sent to the German National Reference Laboratory for *Trichinella* for molecular investigation, which was performed as described previously (Langner et al., 2022).

## 3 Results

**Fig. 1:**
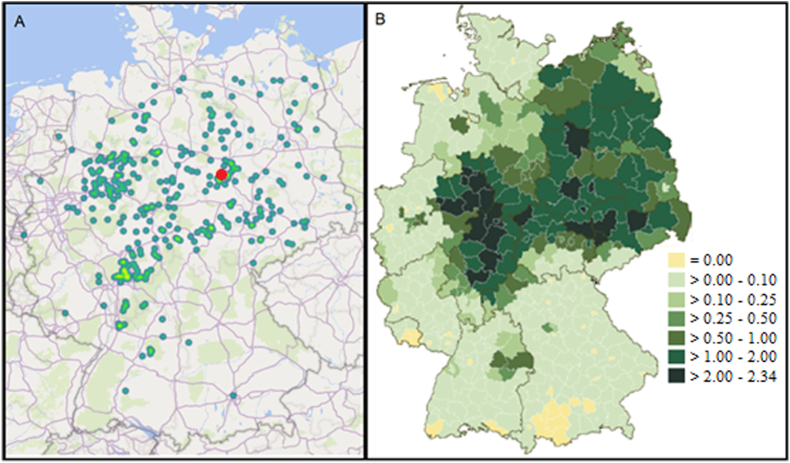
A: Origin of animals used for this study and the origin of the *Trichinella*-like infected individual (red dot). B [reproduced from Hagag et al. (2022)]: average yearly regional hunting bags during seasons 2014/15 to 2017/18 (harvested Individuals/100 ha). All regions with high regional hunting bags are represented in this study. (For interpretation of the references to colour in this figure legend, the reader is referred to the Web version of this article.)

The 904 raccoons included in the study (i.e., 694 males, 199 females, 11 of unknown sex, 876 adults, 26 juveniles, 2 of unknown age) came from different geographical areas as reported in Fig. 1 a. For 109 individuals no information about the origin was available. *Trichinella* spp. was not found in any of the investigated animals.

**Fig. 2:**
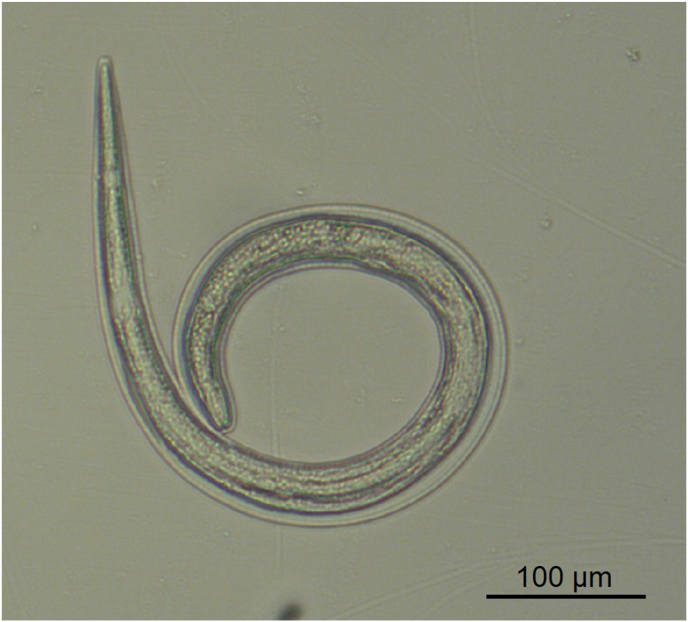
*Trichinella*-like nematode larva: several hundreds of these larvae occurred during trichinella investigation.

**Fig. 3:**
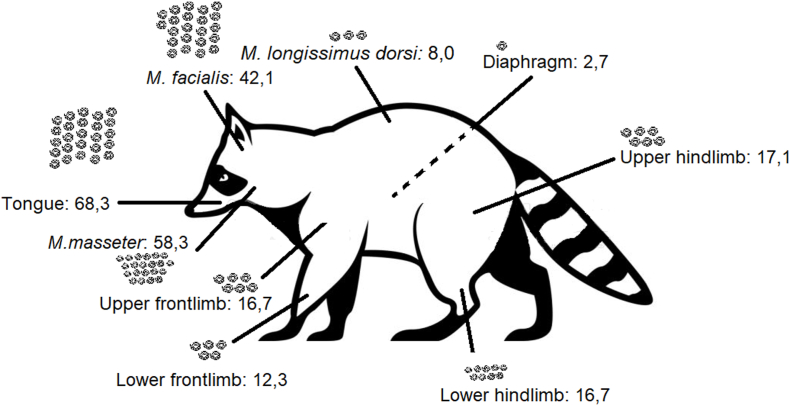
Abundance of unidentified nematode larvae at investigated muscle sites of one individual (larvae per gram). Density varies strongly between different muscles.

**Fig. 4:**
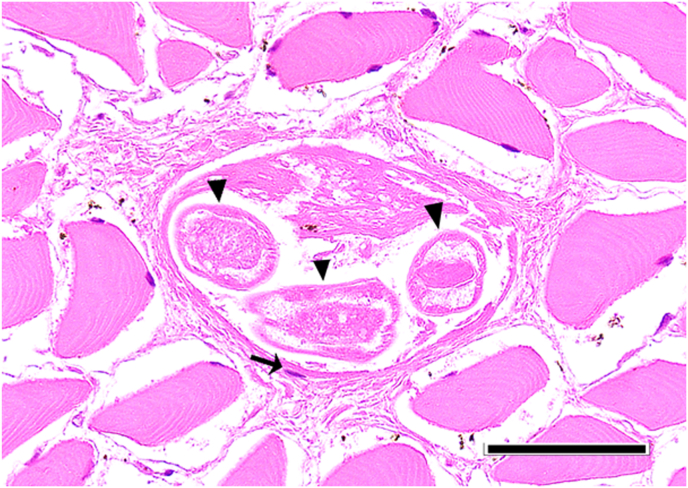
Fig. 5. Masseter. HE. Bar = 50 μm. Parasitic cyst located within skeletal musculature containing two cross- and one longitudinal section of nematode larvae (arrowheads). Note the presence of a compressed, flattened, presumable myocyte nucleus (arrow) adjacent to the cyst wall.

**Fig. 5:**
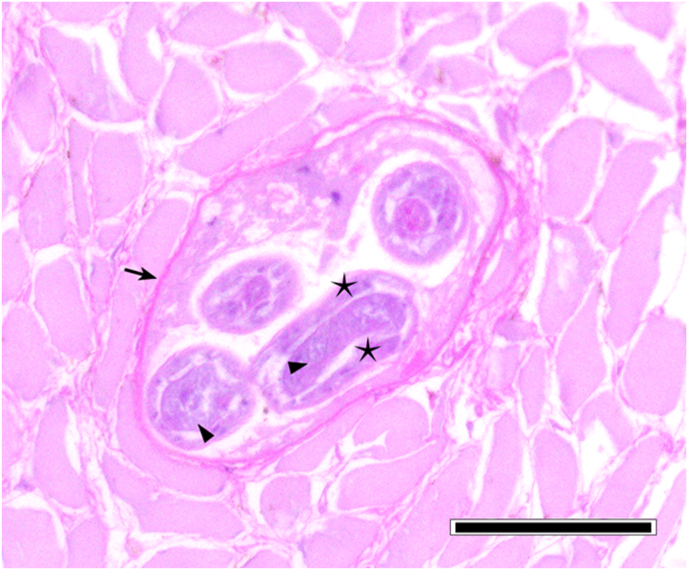
Tongue. PAS. Bar = 50 μm. Parasitic cyst in the skeletal musculature, containing three cross- and one longitudinal section of nematode larvae. Note the PAS-positive cyst wall (arrow) and the presence of bilateral hypodermal structures (stars) and a central degenerated digestive tract (arrowheads).

**Fig. 6:**
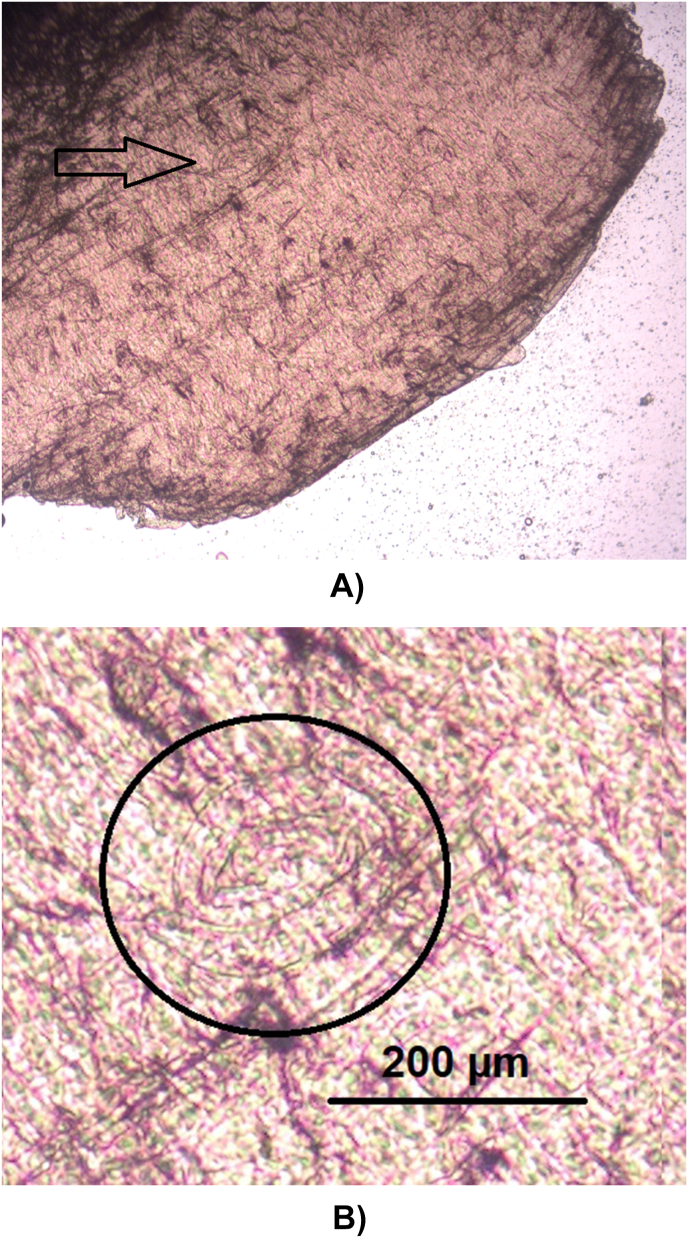
Larvae in striated musculature of raccoon by trichinoscopy. Note the absence of a visible capsule (Original magnification A: 25 x).

However, in one adult female individual, 137 larvae of nematodes were found (Fig. 2). This animal was hunted in February 2020 in Aschersleben, Saxonian-Anhalt at the lake “Königsauer See”. The follow up investigation of the remaining material revealed over 1000 further larvae in varying densities in different muscle sites (Table 1/Fig. 3). The size of these larvae was approximately 660 μm, the shape was similar to *Trichinella*, but the internal tissues were not clear and a stichosome not evident. PCR failed to amplify the selected molecular markers for *Trichinella*. Muscles of this individual were investigated histologically. Despite of freeze/thaw-related artefacts it was possible to detect several cystic structures measuring between 164 x 108 and 110 × 55 μm within all analyzed skeletal muscle samples (Fig. 4, Fig. 5). These structures were encircled by an up to 10 μm thick eosinophilic wall, which stained faintly in a Masson's Trichrome- and PAS special stain (Fig. 5). They contained up to six cross and longitudinal sections of nematode larvae and weakly eosinophilic, unstructured material. Larvae measured approximately 25 μm in diameter, displayed a 1 μm thick, eosinophilic, PAS-positive cuticle. Their pseudocoelom contained poorly preserved hypodermal tissue (4 μm thick, presumably hypodermal bands or wide lateral chords) and a digestive tract (Fig. 5). In some locations a compressed, flattened nucleus adjacent to the cyst wall was present. At the trichinoscopic examination several clear spiral structures were evident in the samples (Fig. 6 a, b).

**Table 1:** Amount of unidentified larvae per gram found in different body sites of one infected raccoon.

Muscle site	mass of sample (g)	counted larvae	larvae/gram
Diaphragm	9,3	25	2,7
Tongue	3,6	246	68,3
lower hindlimb	12,2	298	24,4
lower frontlimb	25	307	12,3
upper hindlimb	15,3	261	17,1
upper frontlimb	17,4	291	16,7
*M. longissimus dorsi*	16,4	132	8,0
*M. facialis*	12,5	526	42,1
*M. masseter*	4,6	268	58,3

**Fig. 7:**
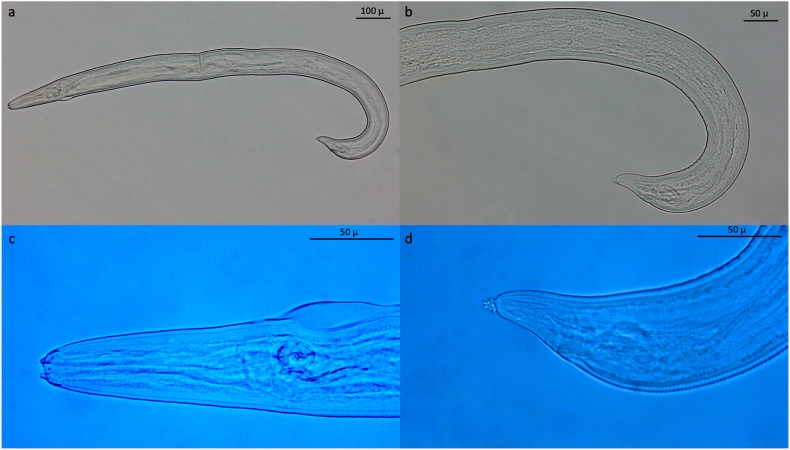
L 3 of *Spirocerca lupi*: These larvae occurred as accidental findings.

In 12 pools nematodes with the following morphological characteristics were found on the 180 μm sieve: Larvae had a slender body, with cuticle slightly transverse with evident striations, anterior end rounded bearing two cephalic oral papillae arranged laterally (Fig. 7). In particular, the mouth orifice was small, and the buccal cavity presented a cylindrical shape. The muscular (short) and the glandular portion (long) of the esophagus were clearly distinct from one another. The intestine presented distinct cells. The tail end presented a rosette-shaped button bearing a group of cuticular spines. Rudimental genitalia and excretory pore were not evident. According to the morphological features, the nematodes were identified as L3 of *Spirocerca lupi*.

## 4 Discussion

In this large epidemiological study *Trichinella* spp. was not detected in raccoons in Germany, with the exception of larvae of an unidentified *Trichinella*-like nematode with intramuscular localization in several sites of a single raccoon. In addition, the detection of *S. lupi* in some individuals suggests that this invasive animal species is a proper definitive host for this parasite in Germany. It is generally accepted that the EU reference method shows significant weakness in detecting dead *Trichinella* larvae (Gamble, 1999; Franssen et al., 2014). As the samples had been frozen before, we used the sequencing sieving method which shows higher sensitivity for dead larvae to minimize this bias. Some important issues should be considered to improve the performance of *Trichinella* detection. Firstly, the amount of *Trichinella* larvae passing through the 64 μm sieve is negligible (Gamble, 1999; Franssen et al., 2014), therefore we suggest to use 64 μm sieve as the smallest one. Due to contamination of the samples, the 180 μm sieve was used to separate the coarse detritus from the finer one. As it cannot be ruled out that dead *Trichinella* spp. larvae remain in this sieve, the content was investigated as well. Considering the uncertainties regarding the predilection site, which is not determined in raccoons, the mass of examined samples should be increased. Based on investigations in canids such as red foxes (*Vulpes vulpes*), arctic foxes (*V. lagopus)* (Kapel et al., 1994, 1995) and raccoon dog (*Nyctereutes procyonoides*) (Mikkonen et al., 2001; Cybulska et al., 2019), the carpal flexor muscle is generally considered a predilection site in carnivores (Pozio and Rossi, 2008). On the other hand, muscle larvae were found only in one out of six forelimbs from infected raccoons (Cybulska et al., 2019). While this study indicates the diaphragm as predilection site (5/6), the results of Kobayashi et al. (2007) contradict this, as they did not find any larvae in diaphragms of six infected individuals. In this study however the tongues showed highest larval burdens, a finding also observed by Snyder et al. (1993). Due to bad digestibility of raccoon meat (Munscher, 2006), we extended the digestion time to 120 minutes, since it has been shown that longer digestion time does not reduce the sensitivity of this method (Li et al., 2010). It was hypothesized that the role of the raccoon in the sylvatic cycle of *Trichinella* spp. could be underestimated due to a lack of studies, as indicated by reports detecting antibodies in muscle juice samples from Germany (Stope, 2019; Cybulska et al., 2020) and the finding of a positive one in a relatively small number of investigated animals (Langner et al., 2022). Contrarily, the results of the present study contradict the above reports. However, in muscle samples of one raccoon, nematodes were found in high abundance by the digestive method. Several aspects are suggestive for the genus *Trichinella*:

i) larvae lay coiled in thin fibrogenic capsules (Fig. 4, Fig. 5) within striated muscles. These are typical findings in infestations with *Trichinella* spp. (Sukura et al., 2002; Pozio et al., 2020).
ii) the collagen capsule surrounding the larva and the absence of evidence for a cellular inflammatory tissue reaction, within the histologically investigated samples, is in accordance to observations of *Trichinella*-infections, at a late stage (Vega-Sánchez et al., 2021).
iii) cysts were found in every investigated muscle sample in different densities (Table 1; Fig. 3) indicating a systemic spread.

On the other hand, following aspects contradict this:

i) morphological aspects such as the absence of stichiform esophagus and an external shield clearly rule out *Trichinella* spp.
ii) the size is too small for known *Trichinella-*species.
iii) the negative result in molecular biological investigation.

Due to advanced tissue degeneration, it was not possible to determine whether or not the cysts are located intracellular. The presence of a compressed, flattened nucleus adjacent to the cyst wall is indicative for an intracellular (most likely intra sarcoplasmatic) localization, which is considered exclusive for *Trichinella* (Eckert and Ossent, 2006; Patra and Sarkar, 2014). The negative result of PCR on the other hand is contraindicative, although the storage condition of the samples, which underwent several freeze and thaw cycles must be considered as unbeneficial. Noteworthy, in several studies larvae morphologically identified as *Trichininella* spp. could not be determined to species level, due to negative results in PCR. This was considered to be caused due DNA-damage during freeze and thaw process (Airas et al., 2010; Moskwa et al., 2012; Deksne et al., 2016; Kärssin et al., 2017; Oksanen et al., 2018). There are only few species of nematodes other than *Trichinella* which invade muscle tissues (Eckert and Ossent, 2006). *Toxocara* spp., *Haycocknema* spp., *Baylisascaris* spp. and *Halicephalobus* spp. must be considered, but can be excluded due to morphological characteristics. Importantly, the appearance of a tissue reaction rules out the possibility of a contamination or a post mortem invasion, both characters which should be considered for samples collected under field condition (Marucci et al., 2013; Karadjian et al., 2020).

The finding of *S. lupi* is remarkable as it is the first proof of an autochthonous infection of this parasite in Germany (Rojas et al., 2020). Despite the larval stage herein identified matches with the characteristics of *S. lupi*, the possibility of the occurrence of other *Spirocerca* spp. cannot be ruled out. Indeed, L3 of a recently identified species named *Spirocerca vulpis* (Rojas et al., 2018) has not yet been described; therefore, it is not known whether the larval stages of the latter have similar morphological characteristics with those of *S. lupi*. The above suggests that further studies are needed to clarify these gaps on *Spirocerca*spp. found in raccoons. Indeed, raccoon is known as potential host of *S. lupi* being detected in individuals from Poland close to the German boarder (Popiołek et al., 2011), from Azerbaijan (Azizova, 2015) and Iran (Sharifdini et al., 2020). Due to the wide diet spectrum of vertebrates and invertebrates (i.e., intermediate host as well as paratenic hosts of *S. lupi*) (Bartoszewicz et al., 2008), this species is potentially highly exposed to this parasite. Instar larvae were all L3, which is a finding already reported in canine definitive hosts, specifically at the dissection of an experimentally infected dog, four days post infection (Sen and Anantaraman, 1971). This might suggest that the larvae of *S. lupi* do not undergo the usual development, but re-encysted as in paratenic hosts (e.g., lizards and small mammals). Therefore, in paratenic hosts L3 can be found in cysts as small white spots in different body sites, including striated muscle (Sen and Anantaraman, 1971). The phenomenon of one host species serving both as main- and paratenic host has been described for other parasites (e.g., for *Alaria alata* in European lynx, *Lynx lynx*) (Zanda Ozoliņa et al., 2020) and might be due to a strategy to complete the biological life cycle of the parasites through the trophic chain of predation (Mendoza Roldan and Otranto, 2023). The method employed for the detection of *Trichinella* larvae is not the most suitable method to detect *Spirocerca* sp. larvae, whose physiological migration leads through gastric wall, the mucosa of the gastric artery wall and the caudal thoracic aorta to the esophagus (Rojas et al., 2020). Yet, there are reports of larvae migrating to other sites in the host's body, such as the thoracic cavity, the nerval system (Chai et al., 2018), subcutaneous tissue or the urinary tract (Rojas et al., 2020). Therefore, though an accidental migration into muscles may occur, the prevalence of 1.3 % may be an underestimation of the actual occurrence of *Spirocerca* sp. in raccoons. Veterinarians should be aware, that this pathogen might also occur as autochthonous infection in central Europe, especially in hunting dogs (Mylonakis et al., 2001) or as incidental finding during *Trichinella*-investigations.

The *Trichinella*-like nematode detected in this study by artificial digestion should be further investigated. The existence of another nematode found in muscle, similar to *Trichinella* spp., should be given particular attention with regard to possible zoonotic potential. This finding and the fact that other studies have already detected *Trichinella* spp. in raccoons underlines the need for raccoons to be tested for *Trichinella* spp. if their meat is intended for human consumption.

## Declaration of competing interest

None.
